# IL-6 stimulates a concentration-dependent increase in MCP-1 in immortalised human brain endothelial cells

**DOI:** 10.12688/f1000research.8153.2

**Published:** 2016-05-11

**Authors:** Jai Min Choi, Odunayo O. Rotimi, Simon J. O'Carroll, Louise F.B. Nicholson

**Affiliations:** 1 Department of Anatomy and Medical Imaging and the Centre for Brain Research, Faculty of Medical and Health Sciences (FMHS), University of Auckland, Auckland, New Zealand

**Keywords:** IL-6 stimulation, systemic inflammation, MCP-1, cell phenotype

## Abstract

Systemic inflammation is associated with neurodegeneration, with elevated interleukin-6 (IL-6) in particular being correlated with an increased risk of dementia. The brain endothelial cells of the blood brain barrier (BBB) serve as the interface between the systemic circulation and the brain microenvironment and are therefore likely to be a key player in the development of neuropathology associated with systemic inflammation. Endothelial cells are known to require soluble IL-6 receptor (sIL-6R) in order to respond to IL-6, but studies in rat models have shown that this is not the case for brain endothelial cells and studies conducted in human cells are limited. Here we report for the first time that the human cerebral microvascular cell line, hCMVEC, uses the classical mIL-6R signalling pathway in response to IL-6 in a concentration-dependent manner as measured by the production of monocyte chemotactic protein (MCP-1). This novel finding highlights a unique characteristic of human brain endothelial cells and that further investigation into the phenotype of this cell type is needed to elucidate the mechanisms of BBB pathology in inflammatory conditions.

## Abbreviations

AD: Alzheimer’s disease; BBB: Blood brain barrier; BD: Becton Dickinson; BSA: Bovine serum albumin; CBA: Cytometric bead array: FACS: fluorescent activated cell sorting; FBS: Fetal bovine serum; gp130: Glycoprotein 130; hCMEC: Human cardiac microvascular endothelial cells; hCMVEC: Human cerebral microvascular endothelial cell; hFGF: human fibroblastic growth factor; HUVEC: Human umbilical vein endothelial cell; IL-6: Interleukin-6; IL-8: Interleukin-8; JAK: Janus kinase; M199: Mediium 199; MCP-1: Monocyte chemoattractant protein-1; mIL-6R: Membrane –bound interleukin-6 receptor; sIL-6r: Soluble interleukin-6 receptor; STAT: Signal transducers and activators of transcription; TNFα: Tumour necrosis factor alpha; VCAM-1: Vascular cell adhesion protein-1.

## Introduction

IL-6 is a pleiotropic cytokine that exerts it effects by binding to an IL-6 receptor, that exists in both a soluble form (sIL-6R) and a membrane-bound form (mIL-6R), that then associates with the membrane-bound glycoprotein 130 (gp130) receptor to initiate intracellular signalling pathways including the Janus kinase/signal transducers and activators of transcription (JAK/STAT) pathway ^1^. gp130 is expressed ubiquitously throughout the body whereas mIL-6R is only expressed in a few cell types including hepatocytes, monocytes, B cells and neutrophils. These cells are able to respond to circulating IL-6 in what is known as classical IL-6 signalling. Cells types such as endothelial cells that do not express mIL-6R are thus dependent on the trans signalling pathway, wherein neutrophils release sIL-6R ^2^. sIL-6R binds to free IL-6 to form a sIL-6R/IL-6 complex which can then interact with gp130 to initiate cell signalling pathways. This has been demonstrated in studies using human umbilical vein endothelial cells (HUVEC) where IL-6 alone did not elicit a response, while the sIL-6R/IL-6 complex induced the production of IL-6, interleukin-8 (IL-8) and monocyte chemotactic protein (MCP-1) ^3^. It appears that brain endothelial cells may however exhibit a different phenotype, with a response to IL-6 alone shown in primary cultures of rat brain endothelial cells ^4, 5^. Such phenotypic differences are of interest as brain endothelial cells not only form a major component of the blood brain barrier (BBB), but elevated levels of circulating serum IL-6 have been correlated with increased risk of developing dementia ^6^. Furthermore, a large meta-analysis of 40 cross-sectional studies found Alzheimer’s disease patients had elevated serum levels of a number of pro-inflammatory cytokines including IL-6 when compared to healthy control subjects ^7^. Since sIL-6 trans-signalling is thought to be pro-inflammatory while the classic mIL-6 signalling is mainly anti-inflammatory ^1^, the nature of the response of brain endothelial cells to IL-6 is very important. We serendipitously found, in a related study, that human brain endothelial cells show a unique response to IL-6. Thus we explored the response of human cerebral microvascular endothelial cells (hCMVEC) following treatment with IL-6, by measuring the concentration of four pro-inflammatory cytokines (IL-6, Il-8, VCAM-1 and MCP-1), in order to better describe the phenotype of these human brain endothelial cells.

## Materials and methods

### hCMVEC cell culture and IL-6 treatment

The human cerebral microvascular endothelial cell (hCMVEC) line was purchased from Applied Biological Materials, Inc. The hCMVEC cell line was cultured in M199 media supplemented with 10% fetal bovine serum (HyClone), 1μg/mL hydrocortisone (Sigma), 3ng/mL hFGF (PeproTech), 10ng/mL hEGF (PeproTech), 10μg/mL heparin (Sigma), 1% GlutaMAX (Gibco), 1% Pen-Strep (Sigma), and 80μM dibutyryl-cAMP (Sigma). All cell culture surfaces were coated with collagen I (Gibco) at a concentration of 1μg/cm ^2^.

For treatment, cells were plated in M199 medium supplemented with 2% fetal bovine serum, 110 nM hydrocortisone, 1% GlutaMAX, 1% Pen-Strep, and 80μM dibutyryl-cAMP for 3 days at which time the media was replaced with media supplemented with varying concentrations of IL-6 (PeproTech) (0.1ng/mL, 1ng/mL, 10ng/mL, and 100ng/mL). 100μL of media was collected at 72 hours post treatment from each well and centrifuged at 420 × G for 10 minutes to remove any cellular debris. 80μL of the supernatant was stored at -80°C until needed.

### Cytometric bead array (CBA)

Soluble IL-6, IL-8, VCAM-1 and MCP-1 were measured by multiplexed cytometric bead array (CBA; BD Biosciences, San Jose, CA, USA; see http://www.bdbiosciences.com/documents/CBA). These were selected as previous studies carried out in our laboratory ^8^ show that they are highly inducible in the hCMVEC cells, whilst having different functions in the inflammatory response: proinflammatory cytokine (IL-6, IL-8), leukocyte adhesion (VCAM-1) and leukocyte recruitment (MCP-1). The CBA was conducted according to the manufacturer’s instructions (except 25 μl of conditioned media was used instead of 50 μl) ^9^. For each cytokine measured, a 10-point standard curve (1–5000 pg/ml) was included. CBA samples were analysed using a BD Accuri C6 Flow Cytometer (BD Biosciences). Data were analysed using FCAP Array software (version 3.0.1, BD Biosciences) which automatically converts the sample mean fluorescent intensity values to pg/ml concentration based on the standard curve.

### Standard preparation

A master cocktail containing the proteins of interest (as supplied by BD Biosciences) at a concentration of 5000pg/mL in CBA assay diluent was prepared. 10 standard solutions of the following concentrations were then prepared using the standard diluent (BD Biosciences): 5000pg/mL, 2500pg/mL, 1250pg/mL, 625pg/mL, 312.5pg/mL, 158.25pg/mL, 78.125pg/mL, 39.0625pg/mL, 19.53125pg/mL and 0pg/mL.

### Bead preparation and addition to samples/standards

The capture beads supplied with the kit for the proteins of interest (CBA Flex set numbers; IL-6, 558276; IL-8, 558277; sVCAM-1, 560427; MCP-1, 558287) were vortexed for 15 seconds and diluted 1:100 in bead diluent to make up the bead cocktail. 25μL of the bead cocktail was added to 25μL of sample/standard in a 96-well plate. The plate was mixed on a shaker at 500rpm for 5 minutes before incubating for 1 hour at room temperature. The detector cocktail was prepared in the same way as the bead cocktail, substituting the capture beads for detector antibodies (as per the BD manufacturer’s protocol) and the bead diluent for detector diluent. 25μL of the detector cocktail was then added to the wells and the plate mixed as before and left to incubate at room temperature for 2 hours. After incubation was complete 200μL of wash buffer (BD Biosciences) was added to each well and the plate centrifuged at 1300rpm for 10 minutes at room temperature. The supernatant was discarded and the beads re-suspended in 50μL of wash buffer before being transferred into a tube for analysis.

### Quantitative analysis

A BD Accuri C6 Flow Cytometer was used to measure the fluorescence of the samples/standards. Using the FCAP Array software version 3.0.1, BD Biosciences), the mean fluorescent intensities of the standards were used to generate a standard curve for each protein of interest. The mean fluorescent intensities of the samples were measured against the standard curve to give the concentrations of the proteins in pg/mL. This data was exported to Microsoft Excel 2010 (Microsoft Corporation) for processing.

### Statistical analysis

Student’s t-test with two-tailed distribution and correction for two samples with unequal variances was carried out on data obtained from CBA using Microsoft Excel 2010. Results were deemed to be significant if the p-value was less than 0.05.

## Results

**Figure 1.**
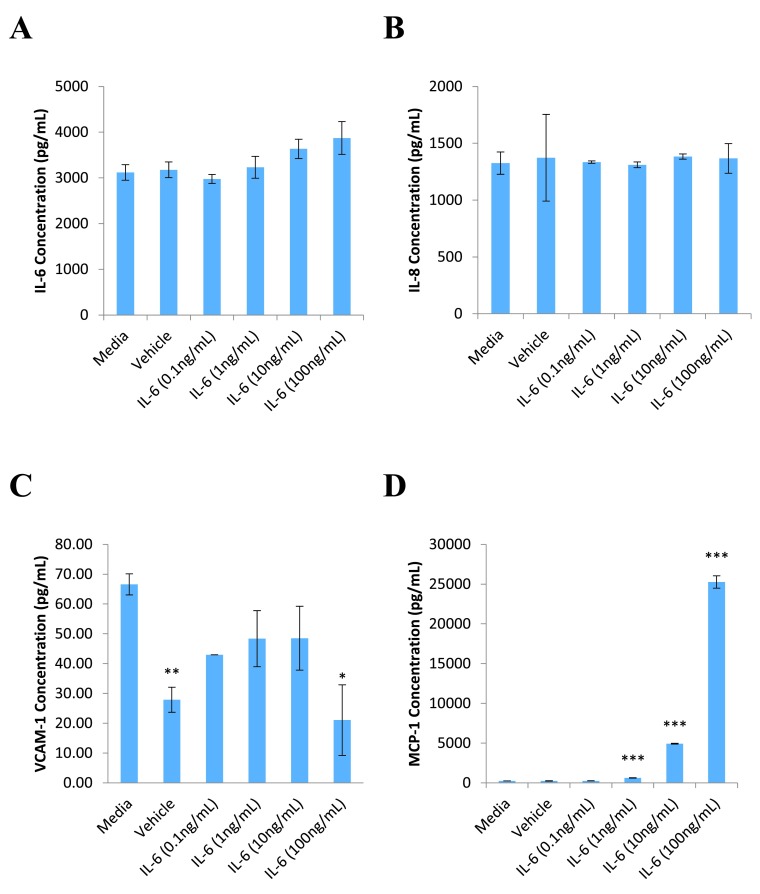
Effect of IL-6 on hCMVEC expression of inflammatory markers. hCMVEC cells were treated with varying concentrations of IL-6 for 72 hours before the media was collected for CBA analysis. The media was assayed for markers of endothelial inflammation IL-6 ( **A**), IL-8 ( **B**), VCAM-1 ( **C**) and MCP-1 ( **D**). Each graph represents the mean ± SD (n=3). Statistical significance was evaluated using Student’s t-test by comparison to the media control; * p<0.05; ** p<0.01; *** p<0.001.

Raw data for effect of IL-6 on hCMVEC expression of inflammatory markers (Figure 1) File shows expression levels of IL-6, IL-8, VCAM-1, and MCP-1 with averages and standard deviations. Click here for additional data file. Copyright: © 2016 Choi JM et al. 2016 Data associated with the article are available under the terms of the Creative Commons Zero "No rights reserved" data waiver (CC0 1.0 Public domain dedication).

hCMVEC cells were shown to be responsive to IL-6 treatment alone ( Figure 1). A clear concentration dependent effect was observed in MCP-1 release, with IL-6 concentrations as low as 1ng/mL producing a statistically significant increase in MCP-1 ( Figure 1D). Such a response was not found for release of IL-6, IL-8 and VCAM-1 ( Figure 1A–C, respectively). Interestingly, no difference was found in IL-6 levels between the media control and treated wells. We also observed that the BSA vehicle caused a decrease in VCAM-1 levels similar to that seen with 100ng/mL IL-6 treatment ( Figure 1C). Given that the scale of release is so small (approximately 70pg/mL) it is difficult to reach a meaningful conclusion about the production of VCAM-1.

## Discussion

To date, the effects of IL-6 on brain endothelial cells have been studied mostly in cells derived from rats, in which IL-6 was found to stimulate eicosanoid production, increase permeability, and increase trans-cellular transport of the human immunodeficiency virus ^4, 5, 10^. This would suggest that rat brain endothelial cells are able to utilise the classical pathway of IL-6 signalling by binding directly to the membrane form of the receptor, mIL-6R. Additionally, *in vivo* studies in rats have shown that deletion of mIL-6R on brain endothelial cells attenuated the development of fever, further supporting the involvement of mIL-6R in pathological processes ^11^. However, such findings in animal models do not always translate into humans. Differences in the signalling pathways of IL-6 between humans and rats have been demonstrated in astrocytes. Human astrocytes were found to require the presence of sIL-6R in order to respond to IL-6 whereas rat astrocytes were able to response to IL-6 alone ^12^. It is not yet known if similar differences exist between human and rat brain endothelial cells. There are few studies examining the response of human brain endothelial cells to IL-6, mostly focusing on permeability ^13, 14^. Nor are there any available studies investigating the immune-modulatory effects of IL-6 in human brain endothelial cells. We have conclusively shown chemokine production in response to IL-6 alone in a human-derived brain endothelial cell line. We found that IL-6 treatment alone resulted in a concentration-dependent release of MCP-1 in hCMVEC cells but did not induce production of IL-6, IL-8 or VCAM-1. The fact that hCMVEC cells respond to IL-6 in the absence of the addition of sIL-6R suggests that hCMVEC cells may be expressing mIL-6R. Future studies confirming the presence of mIL-6R on the surface of hCMVEC cells (or demonstrating the absence of sIL-6R in the system) would add strength to this suggestion. That, notwithstanding, these preliminary results open the unexpected possibility that human brain endothelial cells may be able to utilise both the classical (mIL-6R-mediated) and trans (sIL-6R-mediated) pathways of IL-6 signalling through the presence of mIL-6R and the possible proteolytic cleavage or differential mRNA splicing to form sIL-6R. Furthermore, the lack of IL-6 and IL-8 induction in the hCMVEC response to IL-6 compared to the HUVEC response to the sIL-6/IL-6 complex ^3^ suggests that signalling through mIL-6R and sIL-6R elicits different responses. A possible explanation for this can be found in hepatocytes, where the expression of gp130 is much higher than mIL-6R so that only a small proportion of gp130 receptors will be activated with IL-6 stimulation, whereas the sIL-6R/IL-6 complex can activate all gp130 receptors on the cell resulting in a much stronger effect ^1^. An investigation of the different inflammatory profiles, including pro versus anti-inflammatory events, following activation of either mIL-6R or sIL-6R in hCMVEC cells would be an excellent complementary study to that presented here. Our finding that hCMVEC cells show a concentration-dependent increase in MCP-1 in response to IL-6 is important, as MCP-1 has been shown to be involved in increasing BBB permeability in an *in vitro* co-culture model of rat brain endothelial cells and astrocytes ^15^. The production of MCP-1 by brain endothelial cells helps to explain the effects of IL-6 on BBB permeability.

It is worth noting that the IL-6 concentration was similar between IL-6 treatment groups and control groups. One might expect that the higher concentration treatment groups would have a higher concentration of IL-6 since a greater amount of IL-6 was added. It would be interesting to know if the added IL-6 was internalised by the cells via receptor-mediated endocytosis, as is known to occur in hepatocytes ^16^. While IL-6 is rapidly internalised, the sIL-6R/IL-6 complex undergoes very little internalisation prolonging its signalling capabilities, and adding to the differences between sIL-6R-mediated signalling and mIL-6R-mediated signalling ^1^.

In BB19 cells, another immortalised brain endothelial cell line, VCAM-1 production is reported to be increased 2-fold compared to baseline after 8 hours of IL-6 stimulation. This then declines 24 hours post treatment ^17^. While it is possible that we simply did not observe an increase in VCAM-1 production after IL-6 stimulation it had declined back to baseline by our 72 hour time point, our 72 hour time point was chosen specifically as an earlier study from our laboratory ^8^ has shown that hCMVEC cells produce substantial amounts of many inflammatory markers at 72 hours post treatment with TNF α in comparison to 24 and 48 hours post-treatment. We also found that our BSA vehicle caused a significant decrease in VCAM-1. The amount of BSA in the vehicle treatment group is equivalent to that of the 100ng/mL IL-6 treatment group raising the possibility that the decreased VCAM-1 production with 100ng/mL IL-6 treatment might be due to the BSA. Although there is a study documenting the inhibitory effect of BSA on TNFα-induced endothelial VCAM-1 production ^18^, these authors used much higher concentrations of BSA and only measured the decrease in relation to the induced levels of VCAM-1, not basal levels. Despite the short comings of our study, the findings are significant and pave the way for future studies to further develop this research. For example, HUVEC could be included as a control, specific receptor blocking antibodies used to test the specificity of IL-6 supplementation and repeating this work using microvascular cells of a different origin (for example, human cardiac microvascular endothelial cells; hCMEC) would expand this study and thus provide further evidence supporting the proposal of a unique human brain endothelial cell phenotype.

## Conclusion

There is a paucity of studies investigating the effects of IL-6 in human brain endothelial cells. Our study is the first to report findings suggesting that the human cerebral microvascular cell line, hCMVEC, responds to IL-6 in a way that it is unlikely to be through the soluble sIL-6R but instead uses the classical mIL-6R signalling pathway in a concentration-dependent manner as measured by the production of monocyte chemotactic protein (MCP-1). This is in contrast to previous studies in HUVEC cells that do not respond to IL-6 in the absence of sIL-6R. Thus there is no doubt that hCMVEC cells show a different immunoreactive phenotype to other endothelial cells. This highlights the heterogeneity in immunoreactive phenotypes among endothelial cell subtypes. The response of human brain endothelial cells to pro-inflammatory molecules should be further explored to elucidate the mechanisms of BBB pathology in inflammatory diseases such an Alzheimer’s disease.

## Data availability

The data referenced by this article are under copyright with the following copyright statement: Copyright: © 2016 Choi JM et al.

Data associated with the article are available under the terms of the Creative Commons Zero "No rights reserved" data waiver (CC0 1.0 Public domain dedication). 

*F1000Research*: Dataset 1. Raw data for effect of IL-6 on hCMVEC expression of inflammatory markers ( Figure 1), 10.5256/f1000research.8153.d114985 ^19^
