# Supplementary material for: IL-6 stimulates a concentration-dependent increase in MCP-1 in immortalised human brain endothelial cells
Source: F1000Res. 2016 May 11;5:270. Originally published 2016 Mar 3. [Version 2] doi: 10.12688/f1000research.8153.2 (PMC4900293; doi:10.12688/f1000research.8153.2)
Supplement: Peer review history

# Peer review: Referee response for version 2

**DOI:** 10.5256/f1000research.9413.r13974

**Published:** 2016-06-06

**Authors:** Mansi R. Khanna

**Affiliations:** 1 Center For Neurodegenerative Disease Research, University of Pennsylvania, Philadelphia, PA, USA

The authors have attempted to determine the phenotype of human brain endothelial cells in response to a pro-inflammatory cytokine IL-6.  This study is of significance given the paucity of data in this regard. The data on the induction of MCP-1 in response to IL-6 are compelling. However, I have the following suggestions/questions to lend credibility to some of the claims the authors make:

1.  Even though the authors have discussed future experiments to study the response of other pro- and anti-inflammatory events in the presence of IL-6, this is one of the short-comings of the study. They might want to consider adding some anti-inflammatory molecules to the repertoire. This will also build a case for the type of signaling mechanism in play, ie classical or trans IL-6 signaling.
2. In order to determine the effect of IL-6 alone on the secretion of IL-6, IL-8, VCAM-1 and MCP-1, statistics should be performed to compare all treatment groups to the vehicle. Currently, the effects reported are those of IL-6+vehicle (since comparison has been done to media control). This will be particularly helpful in reaching a meaningful conclusion on the VCAM-1 result.
3. The authors should cite the serendipitous study mentioned in the introduction.  It sounds like it might provide some meaningful background for this piece of work.
4. The vehicle and its concentration must be mentioned in the materials and methods section.

I have read this submission. I believe that I have an appropriate level of expertise to confirm that it is of an acceptable scientific standard, however I have significant reservations, as outlined above.

# Peer review: Referee response for version 2

**DOI:** 10.5256/f1000research.9413.r13788

**Published:** 2016-05-13

**Authors:** Diego Gomez-Nicola

**Affiliations:** 1 CNS Inflammation Group, Centre for Biological Sciences, Southampton General Hospital, The University of Southampton, Southampton, UK

The revised version does not include any of the suggested additional experiments. While I understand that my suggestion of performing a deeper analysis of the inflammatory response could go beyond the scope and format of this research note, I still consider that my first comment, focused to clarifying the mIL6R is a reasonable ask.

The format of this communication would allow the publication of preliminary findings, but I think it is for the author's interest to provide a clear message with their findings, rather than a source of further questions.

I have read this submission. I believe that I have an appropriate level of expertise to confirm that it is of an acceptable scientific standard, however I have significant reservations, as outlined above.

# Peer review: Referee response for version 2

**DOI:** 10.5256/f1000research.9413.r13789

**Published:** 2016-05-11

**Authors:** Robert B Petersen

**Affiliations:** 1 Department of Pathology, Case Western Reserve University, Cleveland, OH, USA

The modification of the discussion adequately addresses previous concerns.

I have read this submission. I believe that I have an appropriate level of expertise to confirm that it is of an acceptable scientific standard.

# Peer review: Referee response for version 1

**DOI:** 10.5256/f1000research.8769.r13376

**Published:** 2016-04-14

**Authors:** Diego Gomez-Nicola

**Affiliations:** 1 CNS Inflammation Group, Centre for Biological Sciences, Southampton General Hospital, The University of Southampton, Southampton, UK

The reported observations are of interest, to understand immune to brain communication. Given the background of the experiments and the potential interest, I have two suggestions of further experimental work that would provide strong support to the authors claims:

1. As acknowledged by the authors, if IL6 alone can elicit a response, this would indicate the potential expression of mIL-6R by hCMVECs. This needs to be confirmed, as its needed to understand the already reported data.
2. The authors comment on the different inflammatory profile caused by activation from mIL-6R or sIL6R. Seems like the current findings just scratch on the surface of that response, and further analysis of pro- vs anti-inflammatory mediators seems a sensible complement to this study.

I have read this submission. I believe that I have an appropriate level of expertise to confirm that it is of an acceptable scientific standard, however I have significant reservations, as outlined above.

# Response

**Published:** 2016-04-18

**Authors:** Louise Nicholson

**Affiliations:** University of Auckland, New Zealand

We thank the reviewer for the constructive feedback and are pleased that our observations add to an understanding of the immune to brain communication.  We accept that these preliminary findings need to be explored further and agree that the two suggested experiments are very valuable indeed in confirming the expression of mIL-6R and the inflammatory profile resulting from the activation of mIL-6R or sIL-6R.  We shall definitely include these recommendations in the discussion of our revised manuscript as a way of advancing these preliminary findings.  

We selected the category of a ’Research note’ for publication of our observations because they are preliminary and do indeed ‘just scratch on the surface’.  We believe however, that the observations are important and of value to the field paving the way for future studies as suggested by the reviewer. At this stage we are not in a position to undertake any further experiments ourselves but would be delighted if someone else picked up the challenge.

# Peer review: Referee response for version 1

**DOI:** 10.5256/f1000research.8769.r12723

**Published:** 2016-03-11

**Authors:** Robert B Petersen

**Affiliations:** 1 Department of Pathology, Case Western Reserve University, Cleveland, OH, USA

This is an interesting preliminary study on the effect of IL6 on an immortalized human cerebral microvascular cell line. The authors show convincingly that addition of IL6, in the absence of exogenous sIL6R, induces production of MCP-1, a chemotactic protein. This induction is a subset of possible responses shown previously in response to IL1-ß and TNF-alpha by the same group. The result also contrasts with an earlier report on the response of HUVEC cells in which the authors showed that the response required sIL6R (the cell culture conditions were quite different than the present study).

The report would be stronger if the authors had showed either the presence of mIL6R on the cell surface or demonstrated the absence of sIL6R in the system.

I have read this submission. I believe that I have an appropriate level of expertise to confirm that it is of an acceptable scientific standard.

# Response

**Published:** 2016-03-15

**Authors:** Louise Nicholson

**Affiliations:** University of Auckland, New Zealand

Thank you for your very positive and constructive feedback. We shall definitely include a sentence or two in our manuscript acknowledging the benefits of future work to confirm the presence of mIL-6R on the surface of our hCMVEC or the absence of sIL-6R in the system we have used.
